# Research Progress on Lycopene in Swine and Poultry Nutrition: An Update

**DOI:** 10.3390/ani13050883

**Published:** 2023-02-28

**Authors:** Jun Chen, Xuehai Cao, Zhouyin Huang, Xingping Chen, Tiande Zou, Jinming You

**Affiliations:** Jiangxi Province Key Laboratory of Animal Nutrition, Jiangxi Province Key Innovation Center of Integration in Production and Education for High-Quality and Safe Livestock and Poultry, Jiangxi Agricultural University, Nanchang 330045, China

**Keywords:** antioxidant, functional feed additive, lycopene, poultry, swine

## Abstract

**Simple Summary:**

Lycopene is a natural, red-colored pigment that is found in plants and has particularly robust antioxidant activity among the carotenoids. Plenty of research has highlighted its antioxidant, anti-inflammatory, anticancer, and antidiabetic properties. Emerging evidence has promoted the development of lycopene as a natural antioxidant feed supplement to enhance the health status and performance of pigs and poultry. This review aimed to summarize the latest research advances on lycopene in swine and poultry, to deepen our understanding of its biological functions and practical applications in livestock production.

**Abstract:**

Oxidative stress and in-feed antibiotics restrictions have accelerated the development of natural, green, safe feed additives for swine and poultry diets. Lycopene has the greatest antioxidant potential among the carotenoids, due to its specific chemical structure. In the past decade, increasing attention has been paid to lycopene as a functional additive for swine and poultry feed. In this review, we systematically summarized the latest research progress on lycopene in swine and poultry nutrition during the past ten years (2013–2022). We primarily focused on the effects of lycopene on productivity, meat and egg quality, antioxidant function, immune function, lipid metabolism, and intestinal physiological functions. The output of this review highlights the crucial foundation of lycopene as a functional feed supplement for animal nutrition.

## 1. Introduction

Oxidative stress severely threatens the productivity and health status of farm animals, which results in huge economic losses for the livestock industry [1]. In animal production, many factors, such as changes in the environment [2], physiological stages [3,4], and exogenous pathogenic toxins (such as mycotoxins) [5], can cause oxidative stress, thus disturbing the redox balance in animal bodies. Oxidative stress refers to the imbalance between antioxidants and pro-oxidants [6]. The excessive production of reactive oxygen species (ROS) and reactive nitrogen radicals (RNS) will cause irreversible damage to cell lipids, proteins, and DNA, thus affecting the physiological functions and production performance of the animals [7]. Antioxidation is defined as the process of antioxidant defense against oxidation in organisms [8]. The antioxidant substances in the body are mainly divided into two categories; one is synthesized by the body itself, and the other is obtained from food [9]. When animals are in special circumstances such as high temperatures, weaning, pregnancy, and so on, the supplementation of exogenous antioxidant substances (such as plant-derived polyphenols and carotenoids) can effectively alleviate the oxidative stress status in animals, reduce oxidative damage, and improve their health and production performance [10,11]. In addition, antibiotic resistance and residues have adversely affected animal production, human health, and environmentally sustainable development [12]. Since the European Union (EU) banned the use of antibiotics as feed additives in animal feed in 2006, researchers have been trying to explore plant-derived feed supplements as safe antibiotic alternatives [13]. After July 2020, China officially entered a new era of in-feed antibiotic bans in livestock production [14]. Therefore, it is urgently necessary to develop natural, green, and safe antibiotic alternatives for the swine and poultry industry.

Lycopene (C_40_H_56_), a red-colored carotenoid, is a natural pigment found in plants, mainly in fruits and vegetables such as tomatoes, carrots, watermelon, and guava [15]. Lycopene has the greatest antioxidant potential among carotenoids due to its chemical structure [16]. Structurally, it contains 2 non-conjugated double bonds and 11 conjugated double bonds [17]. Lycopene has been listed as a nutrient and food additive in more than 50 countries and is widely used in healthy foods, medicine, cosmetics, agriculture, and other fields [18]. Lycopene is a powerful antioxidant, which is the fundamental basis for its health-promoting effects, including anti-inflammatory, anticancer, and antidiabetic potential [19], cardiovascular-protecting abilities [20], and neurobiological, antihypertensive, and anti-aggregative effects [21]. Interestingly, there is an ever-increasing body of evidence indicating that lycopene could be developed as a functional feed additive for swine and poultry. In detail, lycopene has been reported to improve productivity, meat quality, egg quality, antioxidant, immunity, lipid metabolism, and intestinal physiological functions [22,23,24,25]. The biological functions of lycopene are primarily due to its chemical structure [16]. In this paper, we first briefly introduce the sources, physicochemical properties, digestion and absorption, and biological functions of lycopene. Then, we systematically summarize the latest research progress of lycopene in swine and poultry over the last ten years (2013–2022).

## 2. An Overview of Lycopene

### 2.1. The Sources of Lycopene

Lycopene was first discovered as a red pigment in tomatoes by Millardet in 1876; 27 years later, it was named lycopene by Schunck in reference to the scientific name of tomato (*Lycopersicon esculentum*) [26]. Lycopene is widely distributed in tomatoes and also in red vegetables and fruits, such as carrots, sweet potatoes, pumpkin, watermelons, apricots, papaya, pink grapefruits, pink guavas, and rosehips [27]. Although lycopene exists in a variety of foods, tomato plays a leading role in lycopene-producing sources because it is the main extraction source, as well as the cheapest raw material for lycopene [28]. It has been reported that the global gross production of tomatoes was 180.8 million tonnes in 2019 [29]. Tomatoes are a rich source of lycopene, accounting for 80% to 90% of all carotenoid food content [27]. The extraction techniques of lycopene from raw materials commonly include conventional methods, microwave- and ultrasound-assisted extraction, supercritical fluid extraction, and enzyme-assisted extraction [30]. Most importantly, biosynthetic methods have been developed for large-scale lycopene production using biotechnology [31]. Microbial fermentation is a typical traditional biotechnology in lycopene production [32]. Additionally, modern biotechnology, including genetic engineering, protein engineering, and metabolic engineering, has also been applied to lycopene production [31].

### 2.2. The Physico-Chemical Properties of Lycopene

**Figure 1:**
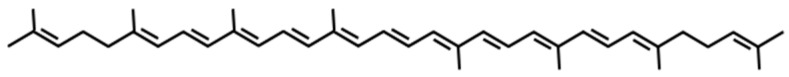
The chemical structure of lycopene.

Lycopene is a form of red-colored carotenoid with the molecular formula of C_40_H_56_ (Figure 1) [28]. It is a polyolefin chain composed of 13 double bonds, 11 of which are conjugated into a linear array (red color factor) [27], making it longer than other carotenoids [33]. Owing to the planar symmetry in its structure, lycopene does not have the same activity as vitamin A [34]. Lycopene is a red waxy pigment, which exists in nature in the form of slender needle-like crystals [33]. It is a liposoluble substance that is insoluble in water and easily soluble in benzene, chloroform, and acetone [27]. In nature, lycopene mostly exists in an all-trans configuration, which is relatively stable in terms of thermodynamics. However, at least 50% of its *cis* isomers are found in the plasma and tissues of humans [33]. The most common forms are 5-*cis*, 9-*cis*, 13-*cis*, and 15-*cis* isomers, which suggests that *cis* isomers are more easily absorbed and utilized by both humans and animals [35].

### 2.3. The Digestion and Absorption of Lycopene

The absorption mode of lycopene is similar to that of lipids, occurring via a passive diffusion pathway [36]. The lycopene in the food matrix is released under the action of gastric acid, bile acid, and enzymes [36]. Upon entering the intestine, lycopene is combined with dietary lipids to form chylomicrons, which are then transported into the mesenteric lymphatic system via diffusion and permeation [37]. Thereafter, the lycopene is finally discharged into portal circulation [36]. This is the main way for lycopene to be absorbed from the gastrointestinal tract [37]. Extrahepatic lipoprotein lipase can partially degrade chyle particles into chyle particle residues. Lycopene and its metabolites are randomly released and transported by low-density lipoprotein (LDL) and very low-density lipoprotein (VLDL) and are finally distributed to the target tissues [35]. The chemical structure of lycopene will affect its distribution process. Through the circulatory system, it will preferentially accumulate in the testis, adrenal gland, liver, and prostate. This uneven distribution indicates its unique biological role in these tissues [38], such as its regulatory role in hepatic lipid metabolism [39].

### 2.4. The Biological Functions of Lycopene

**Figure 2:**
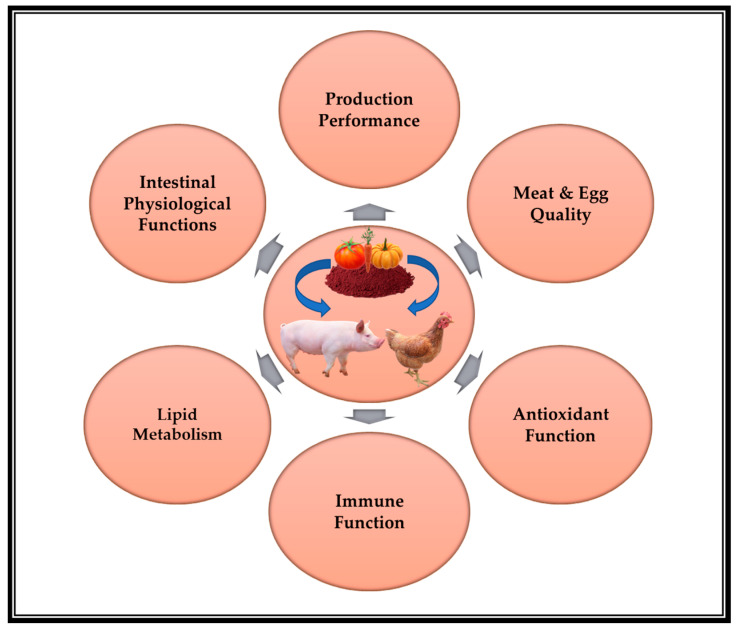
The overview diagram of the beneficial effects of lycopene on swine and poultry.

The unique double-bond structure of lycopene makes it far better than other carotenoids at scavenging free radicals in humans and animals. The greatest antioxidant carotenoid is lycopene [16], followed by tocopherol, carotene, cryptoxanthin, zeaxanthin, β-carotene, and lutein [27]. Lycopene is a robust antioxidant, which is the fundamental basis for its health-promoting effects [40,41]. Furthermore, there is an ever-increasing body of evidence to show that lycopene has anti-inflammatory, anticancer, and antidiabetic potential [19]. Additionally, lycopene has been demonstrated to exert cardiovascular-protecting effects [20], neurobiological effects, and antihypertensive and anti-aggregative effects [21]. Most recently, lycopene has been developed as an effective feed supplement for swine and poultry due to its potent antioxidant potential and red-colored pigment characteristics. The next section of this review will provide a systematic overview of research progress on lycopene in swine and poultry nutrition during the past decade (2013–2022), mainly focusing on its beneficial effects on production performance, meat quality, egg quality, antioxidant function, immune function, lipid metabolism, and intestinal barrier function. Figure 2 presents an overview diagram of the beneficial effects of lycopene on swine and poultry.

## 3. Applications of Lycopene in Swine and Poultry Nutrition during the Past Decade (2013–2022)

### 3.1. Effects on Production Performance

During the last ten years, lycopene has garnered extensive research attention in the field of swine and poultry production. Sun et al. [22] reported that 50 mg/kg dietary lycopene supplementation during gestation and lactation improved the reproductive performance of sows, including increased born-alive piglets, weaned piglets, litter birth weight, litter weaning weight, and decreased born-dead piglets. The authors finally concluded that lycopene promoted sow reproductive performance by regulating milk composition, placental immunity, and antioxidant ability [22]. However, it appears that dietary lycopene inclusion does not influence the production performance of farm animals during the finishing period, especially in the case of finishing pigs. For instance, Fachinello et al. pointed out that dietary addition with a variety of lycopene dosages (12.5, 25, 37.5, and 50 mg/kg) did not impact the growth performance of finishing pigs [42]. Likewise, Fachinello et al. indicated that feeding 12.5, 25, 37.5, and 50 mg/kg of lycopene to finishing pigs did not affect their carcass characteristics or relative organ weights [43]. A recent study conducted by Wen et al. also showed no effects on the performance and carcass characteristics of finishing pigs from dietary lycopene supplementation at 100 or 200 mg/kg [23].

In a poultry study, Sun et al. demonstrated that feeding 40 mg/kg lycopene to Xinghua breeding hens for 35 days increased the fertilization rate and hatchability of their eggs [44]. An et al. also found that the dietary addition of 20 mg/kg lycopene or 1.7% tomato paste for 28 days elevated the egg weight and egg production of Hy-line Brown laying hens [45]. The beneficial additive effect of lycopene on productive performance and carcass characteristics was also observed in Japanese quail, according to a study by Al-Jrrah et al. [46]. In addition, Amer et al. noted that dietary supplementation with lycopene at 300 mg/kg increased the relative growth rate of Japanese quail [47]. In a 42-day feeding trial conducted by Wan et al., supplementation with 10, 20, or 30 mg/kg lycopene increased the average daily gain (ADG), feed conversion ratio (FCR), and final body weight of 1-day-old broilers [48]. Similarly, Wan et al. noticed that broilers supplemented with 100 mg/kg lycopene demonstrated greater body weight at day 21 of the feeding trial [49]. Interestingly, Mezbani et al. revealed that the dietary inclusion of lycopene (50, 100, and 200 mg/kg) enhanced growth performance without affecting the carcass performance of Ross 308 broilers, as indicated by an increased ADG, average daily feed intake (ADFI), and decreased FCR [50]. On the contrary, using tomato paste as a lycopene source, Lee et al. observed no effects on the growth performance and relative organ weights of broilers fed diets containing 10 or 20 mg/kg of lycopene, or 17 g/kg of tomato paste [51].

Most importantly, lycopene has been demonstrated to exert growth-promoting effects on farm animals under stressful conditions, such as heat stress, and due to mycotoxin feed contamination [52,53,54,55,56,57]. For example, Sarker et al. proved that the supplementation of 200 mg/kg lycopene for 42 days increased the ADG and decreased the FCR of broilers under the aflatoxin B1 (AFB_1_) challenge condition [52]. Moreover, the results of Sarker et al. showed that the inclusion of lycopene promoted the growth performance of broilers under the AFB_1_ challenge, as indicated by increased ADG (day_1–21_: 100 mg/kg lycopene; day_22–42_ and day_1–42_: 200 or 400 mg/kg lycopene) and decreased FCR (day_22–42_ and day_1–42_: 200 or 400 mg/kg lycopene) [53]. Under heat stress conditions, the supplementation of 0, 200, and 400 mg/kg lycopene for 42 days has also been confirmed to linearly elevate the growth performance of Ross 308 broilers, as reflected by an increased cumulative feed intake, weight gain, and reduced FCR [54]. It should be noted, however, that the high-dosage supplementation of lycopene (500 mg/kg) has been demonstrated to negatively influence the growth performance of Hubbard broilers, including decreased ADFI and ADG during the first week of the feeding trial [55]. Therefore, in light of the observed results, lycopene (or tomato paste) has great potential to be utilized as a growth-promoting supplement (or feedstuff) for swine and poultry. However, further research is needed to confirm its favorable effects on the production performance of pigs and poultry, using feeding trials with a large population of animals.

### 3.2. Effects on Meat Quality and Egg Quality

Along with rapid economic development and livestock production technology improvement, the ever-increasing attention of consumers has been paid to meat and egg quality in place of meat and egg quantity [58,59]. The quality of meat and eggs is a key criterion for consumers when choosing livestock products [60]. In recent years, lycopene has obtained substantial attention as a natural feed supplement intended to improve meat and egg quality in animal production. As reported by Wen et al., the dietary inclusion of lycopene improved the meat quality of finishing pigs, including reduced L* and b* values, elevated a* value, and intramuscular fat and crude protein contents of the longissimus dorsi (LD) muscle [23]. The thawing loss of the longissimus lumborum (LL) muscle was also found to be linearly reduced when finishing pigs were fed diets supplemented with 0, 12.5, 25, 37.5, and 50 mg/kg of lycopene [43]. Besides this, lycopene was demonstrated to promote muscle fiber type transformation in pigs, which is of great significance in determining meat quality [23]. Specifically, the mRNA levels of *Cytc*, *TFAM*, *TFB1M*, *CS*, *COX1*, *MyHC1*, *MyHC IIa*, *MyHC IIx*, and *TNNI1* were up-regulated, while the mRNA level of *MyHC IIb* was down-regulated in the LD muscle by lycopene supplementation [23]. In addition, the protein expression of slow *MyHC*, *Cytc*, myoglobin, slow-twitch fiber percentage, succinic dehydrogenase (SDH), and malate dehydrogenase (MDH) was increased, while fast *MyHC*, fast-twitch fiber percentage, and lactate dehydrogenase (LDH) activity were decreased in the LD muscle of finishing pigs fed lycopene diets (100 or 200 mg/kg lycopene) [23]. Importantly, the oxidative stability of pork has been reported to be elevated by dietary lycopene feeding (20 mg/kg lycopene, 3.4% tomato paste, or 10 mg/kg lycopene with 1.7% tomato paste) [61]. As we know, lipid oxidation negatively influences the color, nutritional value and flavor, and shelf-life of meat [62]. An et al. reported that the oxidative indices in fresh pork belly meats were decreased when finishing pigs were fed lycopene-supplemented diets, including decreased malondialdehyde (MDA) levels and increased lycopene levels [61]. Likewise, Wen et al. noted that dietary lycopene inclusion improved LD muscle antioxidant status, as indicated by increased total superoxide dismutase (T-SOD) and catalase (CAT) activities, decreased MDA level, up-regulated mRNA levels of *SOD1*, *SOD2*, *CAT*, *GPX1*, *GST*, *GR*, and *Nrf2*, and down-regulated *Keap1* mRNA levels [23]. Similar findings were reported by Correia et al., who observed improved oxidative stability of the LL muscle in young pigs fed 5% tomato pomace for 5 weeks [63]. However, An et al. found that fatty acid composition in the fresh belly meat of finishing pigs was unaffected by dietary lycopene supplementation with 20 mg/kg lycopene, 3.4% tomato paste, or 10 mg/kg lycopene with 1.7% tomato paste in a 28-day feeding trial [61].

Regarding egg quality, the study by Shevchenko et al. showed that supplementing High Line W36 laying hens’ diets with lycopene (20/40/60 mg/kg) for 90 days resulted in improved egg quality, as indicated by an increased carotenoid level and yolk color in fresh eggs or in eggs undergoing a 4 °C and 12 °C storage period [24]. Orhan et al. also indicated that feeding Lohman LSL laying hens with 20 mg/kg lycopene as a purified powder or tomato powder for 84 days improved egg quality, including increased egg weight, yolk color, yolk weight, yolk ratio, yolk lycopene level, and decreased yolk MDA and cholesterol levels [64]. An et al. also found that the dietary addition of 10 or 20 mg/kg lycopene for 28 days increased yolk color and lycopene levels and decreased MDA levels in the eggs of Hy-line Brown laying hens [45]. A previous study by Sun et al. indicated that feeding 40 mg/kg of lycopene to Xinghua breeding hens for 35 days increased the lycopene levels in the serum, eggs, and liver, and an elevated yolk color score [44]. Additionally, Sahin et al. showed that the supplementation of 200 and 400 mg/kg lycopene for 42 days improved the muscle antioxidant status of Ross 308 broilers under heat stress conditions, including a linearly increased lycopene level, glutathione peroxidase (GSH-Px), superoxide dismutase (SOD) activities, decreased MDA level, down-regulated Keap1, and up-regulated Nrf2 protein expression (400 mg/kg lycopene) [54]. Similarly, Lee et al. noticed that dietary supplementation with 10, 20 mg/kg lycopene, or 17 g/kg tomato paste for broilers decreased the thiobarbituric acid-reactive substance (TBARS) value of LDL isolated from broilers at both 2 weeks old and 4 weeks old [51]. However, in laying quails, Hsu et al. suggested that the supplementation of lycopene (6 and 18 mg/kg lycopene as commercial or bacterial lycopene) for 28 days did not affect laying performance or egg quality, or the serum antioxidant status [65]. Therefore, given the current findings, lycopene has huge development value in terms of being utilized as a green feed supplement for meat and egg production, as well as for producing lycopene-enriched meats and eggs as functional foods for humans [66].

### 3.3. Effects on Antioxidant Function

The antioxidant potential of lycopene is one of its predominant biological characteristics, as has been proven in swine and poultry by several animal nutritionists. For instance, Sun et al. demonstrated that dietary 50 mg/kg lycopene supplementation during gestation increased total antioxidant capability (T-AOC) and GSH-Px activity and decreased the H_2_O_2_ and ROS levels in the placental tissues of sows [22]. Additionally, the mRNA levels of *GPX1*, *GPX*4, *FABP4*, *SLPI*, *ANX1*, and *APOE* in the placenta were also up-regulated by lycopene supplementation at 50 mg/kg [22]. In finishing pigs, the dietary supplementation of 0, 12.5, 25, 37.5, and 50 mg/kg of lycopene linearly reduced the TBARS level and increased the 2,2 diphenyl 1 picrylhydrazyl level in the liver [43]. The gene expression of *SOD1* and *CAT* were also found to be linearly affected by dietary lycopene supplementation (0, 12.5, 25, 37.5, and 50 mg/kg) [42]. Similarly, as reported by Wen et al., the dietary inclusion of lycopene improved antioxidant status, including increased T-AOC, T-SOD, GSH-Px, and CAT activities in the serum and liver, and decreased hepatic MDA levels in finishing pigs [23]. Lycopene was also reported to enhance the antioxidant ability and reduce oxidative damage in piglets. In a study conducted by Meng et al., supplementing 50 mg/kg lycopene to weaned piglets for 28 days increased the activities of CAT in serum and SOD in the jejunum, and decreased H_2_O_2_ levels in the serum and jejunum [25]. Lycopene supplementation also up-regulated the mRNA levels of *NRF2*, *SOD2*, *CAT*, *GLUT2*, *GLUT5*, *CD36*, *CLDN1*, and *IL-22*, along with the protein levels of NRF2, CD36, and CLDN1, and down-regulated the mRNA and protein levels of KEAP1 [25]. Those results indicate that lycopene could activate the antioxidant signaling pathway (Keap1/Nrf2) and drive the downstream antioxidant gene expression. Using an in vitro model of a pig embryo, Kang et al. noticed that treating the embryo with 0.1 μM lycopene for 6 days increased the blastocyst formation of embryos, the number of total cells, and the trophectoderm [67]. The authors further investigated the underlying mechanisms and finally concluded that the beneficial effects of lycopene on porcine embryos were attributed to its reducing oxidative stress and apoptosis [67]. In an in vitro model with piglet Sertoli cells, lycopene was also demonstrated to alleviate zearalenone-induced oxidative injury via regulating the Nrf2 signaling pathway [68].

In a broiler-feeding trial by Wang et al., supplementing lycopene (especially at 30 mg/kg) to 1-day-old broilers for 42 days markedly elevated the serum antioxidant status, as indicated by increased T-AOC, GSH-Px, and SOD activities, and decreased MDA level on day 21 of the study [48]. Moreover, the hepatic antioxidant status was also improved by lycopene supplementation, as suggested by increased GSH-Px, SOD activities, decreased MDA levels (10, 20, and 30 mg/kg lycopene) at day 21 of the study, and increased GSH-Px activity (30 mg/kg lycopene), decreased MDA level (10, 20, and 30 mg/kg lycopene) at day 42 of the study [48]. Meanwhile, the Nrf2 pathway was activated by lycopene supplementation, which further drove Nrf2-downstream antioxidant gene expression, including *SOD2*, *NQO1*, and *HO-1* [48]. Feed AFB_1_ contamination commonly occurs in poultry production and seriously threatens the productivity and health of poultry [69]. It has been confirmed that oxidative stress is one of the key mechanisms of AFB_1_ toxicity, and the addition of exogenous antioxidants (such as antioxidant polyphenols and carotenoids) in diets can effectively alleviate the adverse effects of AFB_1_ in poultry [70]. In an experiment by Wan et al., the dietary addition of 100, 200, or 400 mg/kg lycopene all markedly decreased the hepatic activities of cytochrome P450 1A1 (CYP1A1) and cytochrome P450 2A6 (CYP2A6) in broilers fed AFB_1_-contaminated diets in a 42-day feeding trial [71]. The antioxidant status was also enhanced by lycopene supplementation, as suggested by an increased GSH (200 mg/kg lycopene) level, glutathione s-transferase (100 and 400 mg/kg lycopene), glutamine-cysteine ligase, CAT (100 mg/kg lycopene), GSH-Px activities (100, 200 and 400 mg/kg lycopene), decreased AFB1-8,9-epoxide-DNA and ROS levels (100, 200, and 400 mg/kg lycopene), MDA, 8-hydroxydeoxyguanosine (100, 200 and 400 mg/kg lycopene), 4-hydroxynonenal, and protein carbonyl levels (200 and 400 mg/kg lycopene) [71]. Similarly, Sarker et al. documented the finding that feeding 200 mg/kg lycopene for 42 days to broilers fed AFB_1_-contaminated feed elevated the hepatic mitochondrial antioxidant status, including greater mGSH, GSH-Px, MnSOD, and ATP levels, along with lower ROS and H_2_O_2_ levels, and reduced mitochondrial swelling [52]. Additionally, hepatic mitochondrial function was improved, such as increased complex III and complex V, and an up-regulated mRNA level of *MnSOD*, *Trx2*, *TrxR2*, *Prx3*, *PGC-1α*, *NRF1*, and *TFAM* [52]. El-Sheshtawy et al. found that the dietary addition of 100 mg/kg lycopene for 10 days in Pekin ducks decreased the hepatic aflatoxin residue and increased hepatic antioxidant status (increased T-AOC, GST, and CAT activities, and decreased MDA content) [72]. Mezbani et al. indicated that the dietary addition of lycopene improved the serum antioxidant status of Ross 308 broilers, which is suggested by an increase in GSH-Px activity, decreased MDA content (100, 200 mg/kg lycopene), and increased CAT activity (50, 100, 200 mg/kg lycopene) [50]. Sahin et al. observed that dietary supplementation with 200 or 400 mg/kg lycopene for 42 days improved the antioxidant status of Ross 308 broilers under heat-stress conditions, including linearly increased serum lycopene content, GSH-Px, and SOD activities, along with decreased MDA content [54].

In laying hens, as observed by Orhan et al., supplementation of 20 mg/kg lycopene as a purified powder or tomato powder for 84 days decreased serum MDA and cholesterol levels in Lohman LSL laying hens [64]. Likewise, An et al. documented that the dietary addition of 10, 20 mg/kg lycopene or 1.7% tomato paste for 28 days all increased hepatic lycopene levels, while supplementation with 20 mg/kg lycopene or 1.7% tomato paste decreased serum MDA levels in Hy-line Brown laying hens [45]. Sun et al. noted that feeding lycopene to Xinghua breeding hens for 35 days increased the antioxidant status, including increased serum SOD (day_21_, 20, 40, or 80 mg/kg lycopene; day_35_, 80 mg/kg lycopene), T-AOC (day_21_, 20, 40, or 80 mg/kg lycopene), GSH/GSSG (day_21_,_28_,_35_, 20, 40, or 80 mg/kg lycopene), GSH-Px (day_14_, 20, 40, or 80 mg/kg lycopene), and increased hepatic SOD, T-AOC, and GSH/GSSG (day_35_, 20, 40, or 80 mg/kg lycopene) [44]. Amer et al. observed that dietary 300 mg/kg lycopene supplementation increased SOD activity and decreased MDA levels in both the liver and chest muscle of Japanese quail [47]. Sun et al. evaluated the beneficial effects of lycopene (20, 40, and 80 mg/kg) on breeding hens under lipopolysaccharide challenge conditions and observed higher GSH/GSSG in serum at 24 h post-injection of lipopolysaccharide by lycopene feeding at 20, 40, or 80 mg/kg [73]. Therefore, as a natural antioxidant, lycopene could be developed as a functional supplement for swine and poultry. However, along with in vivo feeding trials, more in vitro or ex vivo studies are required to illuminate the specific mechanism of action of lycopene, which is of great importance for promoting the research and application of lycopene in animal nutrition.

### 3.4. Effects on Immune Function

Inflammation is reported to be the cost paid by livestock productivity, and it is the basis for allocating nutrient resources between growth and survival in livestock production [74]. Inflammatory reactions are implicated in animal diseases (such as diarrhea and enteritis), resulting in compromised productivity and higher mortality levels in swine and poultry [75]. Lycopene has been demonstrated to have health-promoting effects on farm animals due to its immune-regulatory functions [56]. Sun et al. highlighted that feeding gestating sows with 50 mg/kg lycopene improved their placental immunity status, including increased secretory immunoglobulin A (sIgA), immunoglobulin G (IgG), immunoglobulin M (IgM) levels, and decreased interleukin-1β (IL-1β), interleukin-8 (IL-8), tumor necrosis factor-α (TNF-α), and interleukin-12 (IL-12) levels [22]. The authors also noticed that the mRNA levels of *IL-1β*, *IL-8*, and *TNF-α* in the placenta were also down-regulated by lycopene supplementation [22]. In a recent study by Liu et al., the dietary addition of 200 mg/kg lycopene for 70 days notably improved the intestinal immune status of finishing pigs, as indicated by decreased IL-1β, TNF-α, and nuclear factor κ-B (NF-κB) levels in the duodenum, down-regulated mRNA levels of *IL-1β* in the jejunum, and up-regulated *interleukin-10* (*IL-10*) mRNA levels in the duodenum and jejunum [76]. Fachinello et al. investigated the impacts of lycopene on the immune responses of finishing pigs and observed that supplementing 0, 12.5, 25, 37.5, and 50 mg/kg lycopene for 28 days linearly increased the plasma albumin and lymphocyte levels, and linearly and quadratically affected neutrophils and the neutrophil/lymphocyte ratio, and also quadratically affected eosinophils in the blood and anti-bovine serum albumin (BSA) production [77].

The immunomodulatory effects of lycopene have also been reported in poultry. In a study conducted by Shevchenko et al., feeding High Line W36 laying hens with lycopene (20, 40, and 60 mg/kg) for 90 days markedly decreased the leukocytes and erythrocytes, but had no effects on the serum antibody titer of hens vaccinated against Newcastle disease, avian rhinotracheitis, egg drop syndrome, and infectious bronchitis [78]. In a 42-day feeding trial by Alwash et al., supplementing the diet with lycopene increased the blood-packed cell volume, heterophils/lymphocytes, and blood hemoglobin level of Japanese quail under heat-stress conditions [79]. Additionally, Sarker et al. revealed that the supplementation of 200 mg/kg lycopene improved the intestinal immunity of broilers under AFB_1_ challenge conditions, as suggested by increased IL-10 levels at day 21 and day 42, and decreased the interferon-γ (IFN-γ) levels in jejunum at day 21 of the study [80]. Furthermore, Sun et al. evaluated the beneficial effects of lycopene on breeding hens under lipopolysaccharide challenge conditions and found that feeding lycopene (20, 40, and 80 mg/kg) to hens for 35 days augmented the immune organ indices of the thymus, spleen, and bursal [73]. However, a high supplemental dosage of lycopene could cause side effects in farm animals. As reported by Pozzo et al., the immune organ weight of the spleen and bursa of Fabricius were reduced by lycopene supplementation at up to 500 mg/kg in the diets of Hubbard broilers [55]. Therefore, further studies are urgently needed to determine the optimal supplemental dosage of lycopene for swine and poultry. Further research is also warranted to clarify the underlying mechanism of action in terms of the immunomodulatory effects of lycopene.

### 3.5. Effects on Lipid Metabolism

Lycopene has been demonstrated to exert modulatory effects on lipid metabolism in swine and poultry. Most recently, Meng et al. reported that supplementing 50 mg/kg lycopene to weaned piglets for 28 days increased their serum total cholesterol (TC) level [25]. In finishing pigs, supplementation with 0, 12.5, 25, 37.5, and 50 mg/kg of lycopene linearly affected plasma cholesterol, high-density lipoprotein (HDL) and LDL levels, and the LDL/HDL ratio of pigs [42]. Wen et al. also proved that the dietary inclusion of lycopene at 100 or 200 mg/kg up-regulated the mRNA levels of *AMPKα1*, *AMPKα2*, *Sirt1*, *PGC-1α*, and up-regulated the protein levels of P-AMPK/AMPK, NRF1, CaMKKβ, Sirt1, PGC-1α in LD muscle of finishing pigs [23]. However, a study by An et al. indicated that serum lipid profiles of finishing pigs were unaffected by dietary supplementation of 20 mg/kg lycopene, 3.4% tomato paste, or 10 mg/kg lycopene with 1.7% tomato paste in a 28-day feeding trial [61]. The placental protein expressions of APOE, ANX1, SLPI, and FABP4 are up-regulated for sows that are fed diets supplemented with 50 mg/kg lycopene during gestation [22].

In broilers, a study by Wan et al. suggested that the dietary inclusion of lycopene at 100 mg/kg for 42 days decreased the abdominal fat weight and abdominal fat percentage of broilers [49]. These results indicated a lipid-lowering property of lycopene. Concurrently, the plasma concentrations of total triglyceride (TG), TC, low-density lipoprotein cholesterol (LDLC), and the hepatic concentrations of fatty acid synthase (FAS) and acetyl-CoA carboxylase (ACC) were reduced in broilers that were fed diets supplemented with 100, 200, or 400 mg/kg of lycopene [49]. Moreover, supplementation of 100, 200, or 400 mg/kg of lycopene all up-regulated the mRNA levels of *AMPK-α*, and down-regulated the mRNA levels of *SREBP-1*, *FAS*, and *ACC* in the livers of broilers, which are indicative of hepatic lipid metabolism [49]. In an experiment by Wan et al., the dietary addition of 100, 200, or 400 mg/kg lycopene for 42 days decreased the levels of aspartate transaminase (AST) and alanine aminotransferase (ALT) in the serum of broilers fed AFB_1_-contaminated diets [71]. A study by Mezbani et al. also indicated that feeding lycopene to Ross 308 increased their glucose and HDL (100, 200 mg/kg lycopene) and decreased their cholesterol, triglyceride, VLDL (50, 100, 200 mg/kg lycopene) levels, also decreasing the hepatic activities of ALT and ALP (100, 200 mg/kg lycopene) [50]. Additionally, Lee et al. revealed that supplementing the diet of broilers with lycopene or 17 g/kg tomato paste decreased plasma TG (10, 20 mg/kg lycopene) and LDL cholesterol (20 mg/kg lycopene) levels at 2 weeks old, and increased the lycopene level in the plasma and liver (10, 20 mg/kg lycopene, 17 g/kg tomato paste) at 4 weeks old [51]. Similar results were found in ducks; the supplementation of 100 mg/kg lycopene for 10 days decreased ALT, AST, gamma-glutamyl transferase (γ-GT), alkaline phosphatase (ALP), and uric acid levels, and increased albumin and total protein levels in the serum of Pekin ducks [72]. However, Pozzo et al. claimed that feeding a high level of lycopene (500 mg/kg) to Hubbard broilers for 35 days decreased the total protein, gamma globulin, albumin, and alpha globulin levels [55].

The regulatory functions of lycopene on fat deposition and lipid metabolism have also been reported in laying and breeding hens. Shevchenko et al. found that feeding High Line W36 laying hens with lycopene (20/40/60 mg/kg) for 90 days markedly increased the serum levels of glucose, creatinine, and ALT, and decreased the serum levels of cholesterol, AST, alkaline phosphatase at day 31 of the study; increased serum levels of glucose, cholesterol, and ALT, and decreased serum level of AST were recorded at day 61 of the study, and increased serum levels of glucose, creatinine, and cholesterol were recorded at day 91 of the study [24]. Additionally, Orhan et al. fed Lohman LSL laying hens with 20 mg/kg lycopene as a purified powder or tomato powder for 84 days and found that lycopene decreased the protein expression of intestinal NPC1L1, MTP, ACAT2, hepatic SREBP1c, ACLY, and LXRα, and increased the protein expression of hepatic ABCG5 and ABCG8 [64]. In a study with quails, Hsu et al. noticed that feeding laying quail with lycopene for 28 days decreased the yolk triglyceride level (6, 18 mg/kg commercial lycopene; 18 mg/kg bacterial lycopene), serum triglyceride level (18 mg/kg bacterial lycopene), and serum total lipid level (6 and 18 mg/kg commercial lycopene) [65]. Under heat-stress conditions, Japanese quail that were fed lycopene diets for 42 days had increased blood glucose and decreased triglyceride levels (with 150, 200, 250, and 300 mg/kg lycopene), increased cholesterol (with 250 and 300 mg/kg lycopene), total protein, and globulin levels (300 mg/kg lycopene) [79]. In contrast, An et al. did not observe alterations in the serum lipid profile of Hy-line Brown laying hens fed 10 or 20 mg/kg lycopene or 1.7% tomato paste diets for 28 days [45]. In breeding hens, Sun et al. highlighted that supplementing lycopene to Xinghua breeding hens for 35 days decreased the total cholesterol (TCHO) levels (day_7_,_35_, 20, 40, and 80 mg/kg lycopene), increased high-density lipoprotein cholesterol (HDLC) (day_35_, 20, 40, 80 mg/kg lycopene), and T3 level (day_14_, 20 mg/kg lycopene) in the serum of hens [44]. Tian et al. demonstrated that feeding lycopene (20, 40, and 80 mg/kg) to Xinghua breeding hens for 35 days regulated the gene expression of fat metabolism. Specifically, lycopene up-regulated the hepatic mRNA levels of *PPARα*, *RARα*, *PGCα* (40 and 80 mg/kg lycopene), *RXRα* (20, 40 and 80 mg/kg lycopene), and down-regulated mRNA levels of *FABP10* (40, 80 mg/kg lycopene), *FABP1* (20, 40, and 80 mg/kg lycopene), and *FATP4* (40 mg/kg lycopene). In the duodenum, the *RARα* mRNA level was up-regulated by the supplementation of 40 or 80 mg/kg lycopene. In the jejunum, lycopene up-regulated the mRNA levels of *PPAPγ*, *RXRγ* (40 and 80 mg/kg lycopene), and *RXRα* (20 and 40 mg/kg lycopene), and down-regulated the mRNA levels of *FATP4* (40 and 80 mg/kg lycopene) [81]. Sun et al. evaluated the beneficial effects of lycopene on breeding hens under lipopolysaccharide challenge conditions and found that lycopene (at 20, 40, and 80 mg/kg) decreased the levels of TCHO, LDLC, and blood urea nitrogen (BUN), and increased HDLC and T3 levels in serum [73]. Therefore, lipid metabolism regulation by lycopene could partially explain the improvement of meat quality, egg quality, and health promotion in swine and poultry. Given the lowering-lipid ability of lycopene, it also has great potential for development as a functional additive to counteract obesity and diabetes in humans.

### 3.6. Effects on Intestinal Physiological Functions

The intestinal tract is the main site of digestion and the absorption of feed nutrients in farm animals and is also an indispensable line of defense against exogenous pathogenic microorganisms and their toxins [82,83,84]. The maintenance of normal intestinal physiological functions and gut homeostasis is a prerequisite for productivity efficiency and well-being in swine and poultry [85,86]. Lycopene has been confirmed to improve the intestinal morphology of pigs, which is indicative of gut health. Most recently, Meng et al. reported that feeding 50 mg/kg lycopene to weaned piglets for 28 days increased villus height (VH) and the ratio of VH to the crypt depth (CD) of the jejunum [25]. The jejunum’s digestive enzyme activities were also increased by lycopene supplementation in piglets, as indicated by elevated lipase, sucrase, maltase, and lactase activities [25]. Consistently, Liu et al. also investigated the beneficial effects of lycopene supplementation on the intestinal morphology of finishing pigs and found that the supplementation of 200 mg/kg lycopene for 70 days significantly augmented the ratio of VH/CD of the jejunum in finishing pigs [76]. In addition, the authors also noticed that the addition of 200 mg/kg lycopene notably up-regulated the mRNA and protein expression of Claudin-1 in the jejunum of pigs, which is indicative of intestinal integrity enhancement via lycopene feeding [76]. The regulatory effects of lycopene on intestinal physiological functions may be attributed to its antioxidant properties. As reported by Liu et al., the supplementation of 100 or 200 mg/kg lycopene for 70 days significantly increased CAT activity in the jejunum and decreased MDA levels in the duodenum of finishing pigs, which suggested that lycopene supplementation promoted intestinal antioxidant capacity [76].

Similar results have been reported in poultry studies. Sarker et al. reported that dietary supplementation with lycopene (at 100, 200, or 400 mg/kg) improved the intestinal morphology of broilers undergoing an AFB_1_ challenge [53]. The authors also observed elevated digestive enzyme activities in the small intestine, including duodenum amylase and lipase activities (day_21_: 200, 400 mg/kg lycopene; day_42_: 200 mg/kg lycopene), and jejunum amylase and lipase activities (day_42_: 200, 400 mg/kg lycopene), as well as ileum amylase activity (day_42_: 200 mg/kg lycopene) [53]. According to the findings of Sarker et al., supplementation with 200 mg/kg lycopene enhanced the intestinal integrity of broilers under AFB_1_ challenge conditions, as suggested by reduced serum diamine oxidase concentrations, with up-regulated jejunum *ZO-1* and *Claudin-1* mRNA levels at day 42 of the experiment [80]. Additionally, Al-Jrrah et al. noted that feeding Japanese quail with lycopene improved their gastrointestinal and organ development [46]. Similarly to the results in swine, Sarker et al. found that supplementing feed with 200 mg/kg lycopene improved the intestinal antioxidant of broilers under AFB_1_ challenge conditions, as suggested by decreased jejunum H_2_O_2_ (day_42_) and MDA levels (day_21_, day_42_), along with increased GSH, GST, and GR levels, as well as up-regulated mRNA levels of *Nrf2*, *HO-1*, *Cu/ZnSOD*, *MnSOD*, and *CAT* (day_42_) [80]. Hence, the maintenance of intestinal physiological functions is another crucial biological function of lycopene for swine and poultry.

A summary of lycopene as a functional feed additive for swine, meat-producing, and egg-producing poultry is presented in Table 1, Table 2 and Table 3, respectively.

## 4. Conclusions

Lycopene is widely considered an effective antioxidant that can capture oxygen free radicals, regulate the cellular expression of related transcription factors, enhance the activities of antioxidant enzymes, and protect cells from free radical damage. As a natural pigment and potent antioxidant, lycopene can be developed as a functional feed supplement for swine and poultry. Several pieces of research have revealed that lycopene has great potential to improve productivity, meat quality, and egg quality, as well as antioxidant activity, immune function, lipid metabolism, and intestinal physiological functions. In view of the current knowledge, lycopene could be supplemented as lycopene products (including commercial lycopene and tomato paste) at 10–400 mg/kg in swine and poultry diets, while a higher supplemental dosage could adversely affect the productivity and health of animals. However, the research on lycopene to alleviate oxidative stress and promote productivity is still in the initial stages, and the research results have not been widely popularized. Therefore, future research directions should further explore the additive effects of lycopene on swine and poultry at different physiological phases and different rearing environments, and, finally, establish the optimum supplemental dosage for the productivity and health of swine and poultry. More importantly, further research is warranted to elaborate the specific mechanism of action of lycopene, which is of great significance in promoting the research and application of lycopene in animal nutrition, or even in human nutrition.

## Figures and Tables

**Table 1:** The summary of lycopene (LYC) as a functional feed additive for swine during the past decade (2013–2022).

Experimental Subject	Supplemental Dosage and Duration	Main Findings	References
Finishing pig	Dosage: 100, 200 mg/kg Duration: 70 days	↑ Jejunum morphology: ↑ VH/CD (200 mg/kg LYC); ↑ Intestinal antioxidant status: ↓ duodenum MDA, ↑ jejunum CAT (all LYC); ↓ Intestinal inflammation: ↓ duodenum IL-1β, TNF-α, NF-κB level, ↓ IL-1β level, and mRNA level in the jejunum; ↑ IL-10 mRNA level in duodenum and jejunum (200 mg/kg LYC); ↑ Intestinal integrity: ↑ mRNA and protein expression of Claudin-1 in the jejunum (200 mg/kg LYC).	[76]
Finishing pig	Dosage: 12.5, 25, 37.5, 50 mg/kg Duration: 75.04–100.45 kg BW	No effects on carcass characteristics and relative organ weights; ↑ Meat quality: linearly ↓ thawing loss of LL muscle; ↑ Antioxidant status: linearly ↓ TBARS, ↑ DPPH in the liver.	[43]
Finishing pig	Dosage: 12.5, 25, 37.5, 50 mg/kg Duration: 28 days	No effects on growth performance; Linearly affected the gene expression of *SOD1* and *CAT*; Linearly affected plasma cholesterol, HDL, LDL, and the LDL/HDL ratio.	[42]
Finishing pig	Dosage: 20 mg/kg LYC, 3.4% tomato paste, 10 mg/kg LYC with 1.7% tomato paste Duration: 28 days	No effects on serum lipid profiles and fatty acid composition in fresh belly meat; ↓ Oxidative indices in fresh pork belly meats: ↓ MDA, ↑ LYC level (all LYC).	[61]
Finishing pig	Dosage: 12.5, 25, 37.5, 50 mg/kg Duration: 28 days	Linearly ↑ plasma albumin level; Linearly ↑ lymphocyte, along with linearly and quadratically affected neutrophils and neutrophil/lymphocyte ratio, and quadratically affected eosinophils in blood; Linearly ↑ anti-BSA production.	[77]
Finishing pig	Dosage: 100, 200 mg/kg Duration: 70 days	No effects on growth performance and carcass characteristics; ↑ Meat quality: ↓ L*, b* values, ↑ a* value, intramuscular fat, crude protein content; ↑ Antioxidant status: serum: ↑ T-AOC, T-SOD, GSH-Px, CAT; liver: ↑ T-AOC, T-SOD, GSH-Px, CAT, ↓ MDA; LD muscle: ↑ T-SOD, CAT, ↓ MDA; LD muscle mRNA level: ↑ *SOD1*, *SOD2*, *CAT*, *GPX1*, *GST*, *GR*, *Nrf2*, ↓ *Keap1*; ↑ Muscle fiber type transformation: mRNA level: ↑ *Cytc*, *TFAM*, *TFB1M*, *CS*, *COX1*, *MyHC1*, *MyHC IIa*, *MyHC IIx*, *TNNI1*; ↓ *MyHC IIb*; protein expression: ↑ slow MyHC, Cytc, Myoglobin; slow-twitch fiber percentage, ↓ fast MyHC, fast-twitch fiber percentage; ↑ SDH, MDH, ↓ LDH activity; Regulated gene expression: ↑ *AMPKα1*, *AMPKα2*, *Sirt1*, *PGC-1α*; Regulated protein expression: ↑ P-AMPK/AMPK, NRF1, CaMKKβ, Sirt1, PGC-1α.	[23]
Sow	Dosage: 50 mg/kg Duration: gestation and lactation	↑ Reproductive performance of sows: ↑ born-alive piglets, weaned piglets, litter birth weight, litter weaning weight, ↓ born-dead piglets; ↑ Placental antioxidant status: ↑ T-AOC, GSH-Px, ↓ H_2_O_2_, ROS; ↑ Placental immunity: ↑ sIgA, IgG, IgM, ↓ IL-1β, IL-8, TNF-α, IL-12; Regulated placental gene expression: ↑ *GPX1*, *GPX*4, *FABP4*, *SLPI*, *ANX1*, *APOE*, ↓ *IL-1β*, *IL-8*, *TNF-α*; Regulated placental protein expression: ↑ APOE, ANX1, SLPI, FABP4; ↑ Milk composition: colostrum: ↑ lactose, IgA, IgG; 10-day milk: lactose.	[22]
Weaned piglet	Dosage: 50 mg/kg Duration: 28 days	↑ Intestinal morphology: ↑ jejunum VH, VH/CD; ↑ Jejunum digestive enzyme activities: ↑ lipase, sucrase, maltase, lactase; ↑ Antioxidant status: ↑ SOD, ↓ H_2_O_2_ (jejunum), ↑ CAT, ↓ H_2_O_2_ (serum); ↑ Serum TC level; Regulated jejunum gene expression: ↓ *KEAP1*, ↑ *NRF2*, *SOD2*, *CAT*, *GLUT2*, *GLUT5*, *CD36*, *CLDN1*, *IL-22*; Regulated jejunum protein expression: ↓ KEAP1, ↑ NRF2, CD36, CLDN1; Regulated gut microbiota.	[25]
Pig embryo	Dosage: 0.1 μM Duration: 6 days	↑ Blastocyst formation of embryos, the number of total cells, and trophectoderm; ↓ Embryos’ ROS levels on day_2_ and day_6_; ↑ JC-1, ↓ cytochrome C fluorescence intensity on day_2_ and day_6_; ↓ Number of CC3-positive cells, apoptosis cells on day_2_ and day_6_; ↓ mRNA level of *SOD1*, *SOD2*, *CAT*, and *BAX/BCL2L1* on day_2_ and day_6_.	[67]
Piglet Sertoli cells (ZEA challenge)	Dosage: 30 μM Duration: 24 h	↑ Cell viability and morphology, ↓ cell apoptosis rate; ↑ Cell antioxidant status: ↑ T-SOD, HO-1, GSH-Px, ↓ ROS, MDA; Regulated gene expression: ↑ *Nrf2*, *GPX1*, *Bcl-2*, ↓ *Keap1*, *LC3*, *Beclin-1*, *Bax*; Immuno-fluorescence: ↑ Nrf2, ↓ LC3; Regulated protein expression: ↑ Nrf2/Histone-H3, Nrf2, HO-1, GPX1, Bcl-2, ↓ keap1, Bax, Beclin-1.	[68]

Abbreviations: ↑, increase; ↓, decrease; AMPKα1, AMP-activated protein kinase α1; AMPKα2, AMP-activated protein kinase α2; ANX1, annexin A1; APOE, apolipoprotein E; Bcl-2, B-cell lymphoma-2; BSA, bovine serum albumin; CaMKKβ, calcium/calmodulin-dependent protein kinase β; CAT, catalase; CC3, cleaved caspase 3; CD, crypt depth; COX1, cyclooxygenase 1; CS, citrate synthase; Cytc, cytochrome c; DPPH, 2,2 diphenyl 1 picrylhydrazyl; FABP4, fatty acid-binding protein 4; GSH-Px, glutathione peroxidase; GPX1(4), glutathione peroxidase 1(4); GR, glutathione reductase; GST, glutathione-stransferase; HDL, high-density lipoprotein; HO-1, heme oxygenase 1; Keap1, Kelch-like ECH-associated protein 1; LD, longissimus dorsi; LDH, lactate dehydrogenase; LDL, low-density lipoprotein; MDA, malondialdehyde; MDH, malate dehydrogenase; MyHC, myosin heavy chain; NF-κB, nuclear factor κ-B; NRF1, nuclear respiratory factor 1; Nrf2, nuclear factor E2-related factor 2; P-AMPK/AMPK, phospho-AMP-activated protein kinase/AMP-activated protein kinase; PGC-1α, peroxisome proliferator-activated receptor g coactivator 1α; ROS, reactive oxygen species; SDH, succinic dehydrogenase; sIgA, secretory immunoglobulin A; Sirt1, sirtuin1; SLPI, antileukoproteinase; SOD(1 or 2), superoxide dismutase(1 or 2); T-AOC, total antioxidant capability; TBARS, thiobarbituric acid-reactive substances; TC, total cholesterol; TFAM, mitochondrial transcription factor A; TFB1M, mitochondrial transcription factor B1; TNNI1, troponin I type 1; T-SOD, total superoxide dismutase; VH, villus height.

**Table 2:** The summary of lycopene (LYC) as a functional feed additive for meat-producing poultry during the past decade (2013–2022).

Experimental Subject	Supplemental Dosage and Duration	Main Findings	References
Arbor Acres broiler	Dosage: 10, 20, 30 mg/kg Duration: 42 days	↑ Growth performance: days_22–42_ and days_1–42_: ↑ ADG, final BW, ↓ FCR (all LYC); ↑ Serum antioxidant status: day_21_: ↓ MDA (all LYC); day_42_: ↑ GSH-Px (all LYC), SOD (20, 30 mg/kg LYC), T-AOC (30 mg/kg LYC); ↑ Liver antioxidant status: day_21_: ↑ GSH-Px, SOD, ↓ MDA (all LYC); day_42_: ↑ GSH-Px (30 mg/kg LYC), ↓ MDA (all LYC); ↑ Hepatic Nrf2-related antioxidant gene expression: day_21_ and day_42_: ↑ *Nrf2* (20, 30 mg/kg LYC), *SOD2* (10, 20, 30 mg/kg LYC), *NQO1* (10, 20, 30 mg/kg LYC), *HO-1* (20, 30 mg/kg LYC, 10 mg/kg LYC for day_21_).	[48]
Arbor Acres broiler (AFB_1_ challenge)	Dosage: 100, 200, 400 mg/kg Duration: 42 days	↓ Serum AST, ALT (all LYC); ↓ Hepatic CYP1A1, CYP2A6 activities (all LYC); ↓ Hepatic AFBO-DNA adducts and ROS levels (all LYC); ↑ Hepatic antioxidant status: ↑ GSH (200 mg/kg LYC), GST (100, 400 mg/kg LYC), GCL, CAT (100 mg/kg LYC), GPx (all LYC), ↓ MDA, 8-OhdG (all LYC), 4-HNE, PC (200, 400 mg/kg LYC).	[71]
Arbor Acres broiler	Dosage: 100, 200, 400 mg/kg Duration: 42 days	↑ Growth performance: ↑ BW at day_21_ (100 mg/kg LYC); ↓ Abdominal fat: ↓ abdominal fat weight (100 mg/kg LYC), abdominal fat percentage (100, 200 mg/kg LYC); ↓ Plasma lipid level: ↓ TG, TC (all LYC), LDLC (100, 200 mg/kg LYC); ↓ Hepatic enzymatic activity: ↓ FAS (400 mg/kg LYC), ACC (all LYC); Regulation of hepatic lipid-metabolism-related gene expression: ↑ *AMPK-α*, ↓ *SREBP-1*, *FAS*, *ACC* (all LYC).	[49]
Arbor Acres broiler (AFB_1_ challenge)	Dosage: 200 mg/kg Duration: 42 days	↑ Growth performance: ↑ ADG, ↓ FCR; ↑ Hepatic mitochondrial antioxidant status: ↑ mGSH, GSH-Px, MnSOD, ATP, ↓ ROS, H_2_O_2_, mitochondrial swelling; ↑ Hepatic mitochondrial function: ↑ complex III, complex V; ↑ mRNA level of *MnSOD*, *Trx2*, *TrxR2*, *Prx3*, *PGC-1α*, *NRF1*, *TFAM*.	[52]
Arbor Acres broiler (AFB_1_ challenge)	Dosage: 100, 200, 400 mg/kg Duration: 42 days	↑ Growth performance: ↑ ADG (day_1–21_: 100 mg/kg LYC; day_22–42_ and day_1–42_: 200, 400 mg/kg LYC), ↓ FCR (day_22–42_ and day_1–42_: 200, 400 mg/kg LYC); ↑ Intestinal morphology: duodenum: ↓ CD, ↑ VH, VH/CD (day_21_,_42_: all LYC); jejunum: ↓ CD (day_21_: all LYC), ↑ VH (day_42_: 200, 400 mg/kg LYC), VH/CD (day_21_: all LYC; day_42_: 200, 400 mg/kg LYC); ileum: ↓ CD (day_21_: all LYC), ↑ VH, VH/CD (day_21_,_42_: all LYC); ↑ Digestive enzyme activities: duodenum: ↑ amylase, lipase (day_21_: 200, 400 mg/kg LYC; day_42_: 200 mg/kg LYC); jejunum: ↑ amylase, lipase (day_42_: 200, 400 mg/kg LYC); ileum: ↑ amylase (day_42_: 200 mg/kg LYC).	[53]
Arbor Acres broiler (AFB_1_ challenge)	Dosage: 200 mg/kg Duration: 42 days	↑ Intestinal immunity: ↓ jejunum IFN-γ (day_21_), ↑ IL-10 level (day_21_, day_42_); ↑ Intestinal integrity: ↓ serum DAO level, ↑ jejunum *ZO-1*, *Claudin-1* mRNA level (day_42_); ↑ Intestinal antioxidant status: ↓ jejunum H_2_O_2_ (day_42_), MDA level (day_21_, day_42_), ↑ GSH, GST, GR level, mRNA level of *Nrf2*, *HO-1*, *Cu/ZnSOD*, *MnSOD*, *CAT* (day_42_).	[80]
Pekin duck (Aflatoxin challenge)	Dosage: 100 mg/kg Duration: 10 days	↓ Hepatic aflatoxin residue; ↑ Hepatic antioxidant status: ↑ T-AOC, GST, CAT, ↓ MDA; Altered serum indices: ↓ ALT, AST, γ-GT, ALP, uric acid, ↑ albumin, total protein.	[72]
Ross 308 broiler	Dosage: 50, 100, 200 mg/kg Duration: 21 days (d_21–42_)	↑ Growth performance: ↑ ADG: day_29–35_ (100, 200 mg/kg LYC), day_36–42_ (all LYC), day_21–42_ (100 mg/kg LYC); ADFI: ↓ day_21–28_ (all LYC), ↑ day_29–35_ (100, 200 mg/kg LYC), day_36–42_ (200 mg/kg LYC), day_21–42_ (all LYC); ↓ FCR: day_21–28_, day_36–42_, day_21–42_ (all LYC); No effects on carcass performance; Altered serum biochemical indices: ↑ glucose, HDL (100, 200 mg/kg LYC), ↓ cholesterol, triglyceride, VLDL (all LYC); ↑ Serum antioxidant status: ↑ GSH-Px, ↓ MDA (100, 200 mg/kg LYC), ↑ CAT (all LYC); ↓ Hepatic enzyme activities: ↓ ALT and ALP (100, 200 mg/kg LYC).	[50]
Ross 308 broiler (heat-stress conditions)	Dosage: 200, 400 mg/kg Duration: 42 days	↑ Growth performance: linearly ↑ CFI, WG, ↓ FCR; ↑ Serum antioxidant status: linearly ↑ LYC, GSH-Px, SOD, ↓ MDA; ↑ Muscle antioxidant status: linearly ↑ LYC, GSH-Px, SOD, ↓ MDA; Activated muscle Keap1/Nrf2 pathway: ↓ Keap1, ↑ Nrf2 protein expression (400 mg/kg LYC).	[54]
Ross broiler	Dosage: 10, 20 mg/kg LYC, 17 g/kg tomato paste Duration: 28 days	No effects on growth performance and relative organ weight; ↓ Plasma TG (10, 20 mg/kg LYC) and LDL cholesterol (20 mg/kg LYC) at 2 weeks old; ↑ Lycopene level in plasma and liver (10, 20 mg/kg LYC, 17 g/kg tomato paste) at 4 weeks old; ↓ TBARS value of LDL isolated from broilers during 60, 90, 120, 180, or 240 min incubation at 2 weeks old (10, 20 mg/kg LYC, 17 g/kg tomato paste), as well as during 60, 120, 180, 240 min incubation at 4 weeks old (10, 20 mg/kg LYC, 17 g/kg tomato paste) except for 90 min (17 g/kg tomato paste).	[51]
Hubbard broiler	Dosage: 500 mg/kg Duration: 35 days	↓ Growth performance: ↓ ADFI, ADG (day_1–8_); ↓ Slaughter performance: ↓ bursa of Fabricius, spleen weight; Altered haemato-biochemical indices: ↓ total protein, gamma globulin, albumin, alpha globulin; Degenerative lesions in the bursa of Fabricius and spleen; No effects on chemical composition and MDA level in the breast and thigh; No effects on oxidative stress in the kidney and liver.	[55]

Abbreviations: ↑, increase; ↓, decrease; ACC, acetyl-CoA carboxylase; ADFI, average daily feed intake; ADG, average daily gain; AFB_1_, aflatoxin B1; AFBO-DNA, AFB1-8,9-epoxide-DNA; ALP, alkaline phosphatase; ALT, alanine aminotransferase; AMPK-α, adenosine monophosphate activated protein kinase α; AST, aspartate aminotransferase; ATP, adenosine triphosphate; BW, body weight; CAT, catalase; CD, crypt depth; CFI, cumulative feed intake; Cu/ZnSOD, copper and zinc superoxide dismutase; CYP1A1(2A6), cytochrome P450 1A1(2A6); DAO, diamine oxidase; 8-OhdG, 8-hydroxydeoxyguanosine; FAS, fatty acid synthase; FCR, feed conversion ratio; 4-HNE, 4-hydroxynonenal; γ-GT, glutamyl transferase; GCL, glutamine-cysteine ligase; GR, glutathione reductase; GPx (GSH-Px), glutathione peroxidase; GSH, reduced glutathione; GST, glutathione-stransferase; HDL, high-density lipoprotein; HO-1, heme oxygenase 1; IFN-γ, interferon-γ; Keap1, Kelch-like ECH-associated protein 1; LDL, low-density lipoprotein; LDLC, low density lipoprotein cholesterol; MDA, malondialdehyde; mGSH, mitochondrial glutathione; MnSOD, manganese superoxide dismutase; NQO1, NAD(P)H quinone dehydrogenase 1; NRF1, nuclear respiratory factor 1; Nrf2, nuclear factor E2-related factor 2; PC, protein carbonyl; PGC-1α, peroxisome proliferator-activated receptor g coactivator 1α; Prx3, peroxiredoxin-3; ROS, reactive oxygen species; SOD(2), superoxide dismutase(2); SREBP-1, sterol regulatory element-binding protein 1; T-AOC, total antioxidant capability; TBARS, thiobarbituric acid-reactive substances; TC, total cholesterol; TFAM, mitochondrial transcription factor A; TG, total triglyceride; Trx2, thioredoxin 2; TrxR2, thioredoxin reductase 2; VH, villus height; VLDL, very low-density lipoprotein; WG, weight gain; ZO-1, zonula occludens-1.

**Table 3:** The summary of lycopene (LYC) as a functional feed additive for egg-producing poultry during the past decade (2013–2022).

Experimental Subject	Supplemental Dosage and Duration	Main Findings	References
High Line W36 laying hen	Dosage: 20/40/60 mg/kg (Day_1–30_/Day_31–60_/Day_61–90,_ different dosages during different phases) Duration: 90 days	↑ Serum metabolic status: day_31_: ↑ glucose, creatinine, ALT, ↓ cholesterol, AST, alkaline phosphatase; day_61_: ↑ glucose, cholesterol, ALT, ↓ AST; day_91_: ↑ glucose, creatinine, cholesterol); ↑ Egg quality: day_30–31_: ↑ carotenoid level and yolk color of fresh eggs and stored eggs (4 °C or 12 °C for 30 days); day_60–61_: ↑ carotenoid level and yolk color of fresh eggs and 4 °C-stored eggs, yolk color of 12 °C-stored eggs; day_90–91_: yolk color of fresh eggs and stored eggs (4 °C or 12 °C for 30 days), carotenoid level of 4 °C-stored eggs.	[24]
High Line W36 laying hen	Dosage: 20/40/60 mg/kg (Day_1–30_/Day_31–60_/Day_61–90_) Duration: 90 days	Hematological indices: ↓ leukocytes (day_61_, day_91_), erythrocytes (day_91_); No effects on serum antibody titer of hens vaccinated against Newcastle disease etc.	[78]
Lohman LSL laying hen	Dosage: 20 mg/kg LYC as a purified powder or tomato powder Duration: 84 days	↑ Egg quality: ↑ egg weight, yolk color, yolk weight, yolk ratio, yolk LYC level, ↓ yolk MDA and cholesterol level (all LYC); ↓ Serum MDA and cholesterol level (all LYC); Regulated cholesterol metabolism: ↓ protein expression of intestinal NPC1L1, MTP, ACAT2, hepatic SREBP1c, ACLY, and LXRα, ↑ hepatic ABCG5 and ABCG8 (all LYC).	[64]
Hy-line Brown laying hen	Dosage: 10, 20 mg/kg LYC, 1.7% tomato paste Duration: 28 days	↑ Laying performance: ↑ egg weight (all LYC), egg production (20 mg/kg LYC, 1.7% tomato paste); ↑ Egg quality: ↑ yolk’ color (10, 20 mg/kg LYC), LYC level, ↓ MDA level (all LYC); ↓ Oxidative stress: ↓ serum MDA level (20 mg/kg LYC, 1.7% tomato paste), ↑ liver LYC level (all LYC); No effects on serum lipid levels.	[45]
Xinghua breeding hen	Dosage: 20, 40, 80 mg/kg Duration: 35 days	Regulated gene expression of fat metabolism: Liver: ↑ PPARα, RARα, PGCα, ↓ FABP10 (40, 80 mg/kg LYC), ↑ RXRα, ↓ FABP1 (all LYC), FATP4 (40 mg/kg LYC); Duodenum: ↑ RARα (40, 80 mg/kg LYC); Jejunum: ↑ PPAPγ, RXRγ, ↓ FATP4 (40, 80 mg/kg LYC), ↑ RXRα (20, 40 mg/kg LYC).	[81]
Xinghua breeding hen	Dosage: 20, 40, 80 mg/kg Duration: 35 days	↑ Production performance: ↑ fertilization rate, hatchability of eggs set (40 mg/kg LYC); ↑ LYC level: ↑ serum, egg (day_7_,_14_,_21_,_28_,_35_, all LYC), liver (day_35_, all LYC); ↑ Egg quality: ↑ Roche yolk color fan (day_7_,_14_,_21_,_28_,_35_, all LYC); ↑ Serum antioxidant status: ↑ SOD (day_21_, all LYC; day_35_, 80 mg/kg LYC), T-AOC (day_21_, all LYC), GSH/GSSG (day_21_,_28_,_35_, all LYC), GSH-Px (day_14_, all LYC); ↑ Hepatic antioxidant status (day_35_): ↑ SOD, T-AOC, GSH/GSSG (all LYC); ↑ Serum lipid metabolism: ↓ TCHO (day_7_,_35_, all LYC), ↑ HDLC (day_35_, all LYC), ↑ T3 (day_14_, 20 mg/kg LYC).	[44]
Xinghua breeding hen (LPS challenge)	Dosage: 20, 40, 80 mg/kg Duration: 35 days	↑ Serum antioxidant status: ↑ GSH/GSSG (0, 24 h post-challenge); ↑ Serum lipid metabolism: ↓ TCHO (0 h post-challenge), ↑ HDLC (0, 6, 24 h post-challenge), ↓ LDLC (6, 24 h post-challenge), ↓ BUN (24 h post-challenge), ↑ T3 (6, 24 h post-challenge); ↑ Organ indices: ↑ thymus, spleen, bursal.	[73]
Japanese quail	Dosage: 100, 200, 300 mg/kg Duration: 28 days	↓ FI (100 mg/kg LYC), RGR (200 mg/kg LYC), ↑ RGR (300 mg/kg LYC); ↑ Antioxidant status: liver: ↓ MDA (200, 300 mg/kg LYC), ↑ SOD (300 mg/kg LYC); chest muscle: ↓ MDA, cholesterol, ↑ SOD (300 mg/kg LYC).	[47]
Japanese quail	Dosage: 50, 100 mg/kg LYC as pure LYC (T_1_, T_2_) or tomato powder (T_3_, T_4_), or red bell pepper powder (T_5_, T_6_) Duration: 49 days	↑ Productive performance: ↑ final BW, BW gain (T_1,2,3,5,6_), production index, ↓ FCR (T_1,2,3,5,6_), economic efficiency index (all LYC); ↑ Carcass characteristic: ↑ carcass weight, gizzard yield (T_5_), liver yield (T_1,3,5,6_); ↑ Gastrointestinal and organ development: ↑ small intestinal weight (T_1,2,4_), duodenum weight (T_2,4_), jejunum and ileum weight (T_2,3_), cecum weight (T_4_), cecum length (T_2,3,4,6_), ↓ abdominal fat weight (all LYC), spleen weight (T_3,4,5,6_), bursa of Fabricius weight (T_1,2,5,6_), bursa index (all LYC).	[46]
Japanese quail (heat-stress conditions)	Dosage: 150, 200, 250, 300 mg/kg Duration: 42 days	↑ Blood packed cell volume, heterophils/lymphocytes (200, 250 mg/kg LYC), ↑ blood hemoglobin level (all LYC); ↑ Blood glucose, ↓ triglycerides level (all LYC), ↑ cholesterol (250, 300 mg/kg LYC), total protein, globulin level (300 mg/kg LYC).	[79]
Laying quail	Dosage: 6, 18 mg/kg LYC as commercial LYC or bacterial LYC Duration: 28 days	No effects on laying performance, egg quality, and serum antioxidant indices; ↓ Yolk triglyceride level (6,18 mg/kg commercial LYC; 18 mg/kg bacterial LYC); ↓ Serum triglyceride level (18 mg/kg bacterial LYC); ↓ Serum total lipid level (6 and 18 mg/kg commercial LYC).	[65]

Abbreviations: ↑, increase; ↓, decrease; ABCG5/8, ATP binding cassette transporters sub-family G member 5/8; ACAT2, Acyl-CoA: cholesterol acyltransferase 2; ACLY, ATP citrate lyase; ALT, alanine aminotransferase; AST, aspartate transaminase; BW, body weight; BUN, blood urea nitrogen; FABP1/10, fatty acid-binding protein 1/10; FATP4, fatty acid transport protein 4; FCR, feed conversion ratio; FI, feed intake; GSH/GSSG, reduced glutathione to oxidized glutathione ratio; GSH-Px, glutathione peroxidase; HDLC, high density lipoprotein cholesterol; LDLC, low density lipoprotein cholesterol; LXRα, liver X receptor alpha; MDA, malondialdehyde; MTP, microsomal triacylglycerol transport protein; NPC1L1, Niemann-Pick C1-Like 1; RARα, retinoic acid receptor α; PGCα, peroxisome proliferator-activated receptor gamma coactivator 1-α; PPARα/γ, peroxisome proliferator-activated receptor α/γ; RGR, relative growth rate; RXRα/γ, retinoid X receptor α/γ; SOD, superoxide dismutase; SREBP1c, sterol regulatory element binding protein-1c; T-AOC, total antioxidant capacity; TCHO, total cholesterol; T3, triiodothyroxine.

## Data Availability

Not applicable.

## References

[1] Chen J., Huang Z., Cao X., Zou T., You J., Guan W. (2023). Plant-derived polyphenols in sow nutrition: An update. Anim. Nutr..

[2] Emami N.K., Jung U., Voy B., Dridi S. (2021). Radical response: Effects of heat stress-induced oxidative stress on lipid metabolism in the avian liver. Antioxidants.

[3] Orengo J., Hernández F., Martínez-Miró S., Sánchez C.J., Peres Rubio C., Madrid J. (2021). Effects of commercial antioxidants in feed on growth performance and oxidative stress status of weaned piglets. Animals.

[4] Chen J., Han J., Guan W., Chen F., Wang C., Zhang Y., Lv Y., Lin G. (2016). Selenium and vitamin E in sow diets: I. Effect on antioxidant status and reproductive performance in multiparous sows. Anim. Feed. Sci. Technol..

[5] Chen J., Huang Z., Cao X., Chen X., Zou T., You J. (2022). Plant-derived polyphenols as Nrf2 activators to counteract oxidative stress and intestinal toxicity induced by deoxynivalenol in swine: An emerging research direction. Antioxidants.

[6] Dobrică E.-C., Cozma M.-A., Găman M.-A., Voiculescu V.-M., Găman A.M. (2022). The involvement of oxidative stress in psoriasis: A systematic review. Antioxidants.

[7] Kim S.W., Weaver A.C., Shen Y.B., Zhao Y. (2013). Improving efficiency of sow productivity: Nutrition and health. J. Anim. Sci. Biotechnol..

[8] Forman H.J., Zhang H. (2021). Targeting oxidative stress in disease: Promise and limitations of antioxidant therapy. Nat. Rev. Drug. Discov..

[9] Ponnampalam E.N., Kiani A., Santhiravel S., Holman B.W.B., Lauridsen C., Dunshea F.R. (2022). The importance of dietary antioxidants on oxidative stress, meat and milk production, and their preservative aspects in farm animals: Antioxidant action, animal health, and product quality—Invited review. Animals.

[10] Li Q., Yang S., Chen F., Guan W., Zhang S. (2022). Nutritional strategies to alleviate oxidative stress in sows. Anim. Nutr..

[11] Hao Y., Xing M., Gu X. (2021). Research progress on oxidative stress and its nutritional regulation strategies in pigs. Animals.

[12] Ghimpețeanu O.M., Pogurschi E.N., Popa D.C., Dragomir N., Drăgotoiu T., Mihai O.D., Petcu C.D. (2022). Antibiotic use in livestock and residues in food—A public health threat: A review. Foods.

[13] Kobayashi Y., Oh S., Myint H., Koike S. (2016). Use of Asian selected agricultural byproducts to modulate rumen microbes and fermentation. J. Anim. Sci. Biotechnol..

[14] Zeng Y., Wang Z., Zou T., Chen J., Li G., Zheng L., Li S., You J. (2021). Bacteriophage as an alternative to antibiotics promotes growth performance by regulating intestinal inflammation, intestinal barrier function and gut microbiota in weaned piglets. Front. Vet. Sci..

[15] Mohammad Azmin S.N.H., Sulaiman N.S., Mat Nor M.S., Abdullah P.S., Abdul Kari Z., Pati S. (2022). A review on recent advances on natural plant pigments in foods: Functions, extraction, importance and challenges. Appl. Biochem. Biotechnol..

[16] Imran M., Ghorat F., Ul-Haq I., Ur-Rehman H., Aslam F., Heydari M., Shariati M.A., Okuskhanova E., Yessimbekov Z., Thiruvengadam M. (2020). Lycopene as a natural antioxidant used to prevent human health disorders. Antioxidants.

[17] Przybylska S., Tokarczyk G. (2022). Lycopene in the prevention of cardiovascular diseases. Int. J. Mol. Sci..

[18] Han G.-M., Liu P. (2017). Higher serum lycopene is associated with reduced prevalence of hypertension in overweight or obese adults. Eur. J. Integr. Med..

[19] Mirahmadi M., Azimi-Hashemi S., Saburi E., Kamali H., Pishbin M., Hadizadeh F. (2020). Potential inhibitory effect of lycopene on prostate cancer. Biomed. Pharmacother..

[20] Costa-Rodrigues J., Pinho O., Monteiro P.R.R. (2018). Can lycopene be considered an effective protection against cardiovascular disease?. Food Chem..

[21] Khan U.M., Sevindik M., Zarrabi A., Nami M., Ozdemir B., Kaplan D.N., Selamoglu Z., Hasan M., Kumar M., Alshehri M.M. (2021). Lycopene: Food sources, biological activities, and human health benefits. Oxid. Med. Cell Longev..

[22] Sun S., Meng Q., Bai Y., Cao C., Li J., Cheng B., Shi B., Shan A. (2021). Lycopene improves maternal reproductive performance by modulating milk composition and placental antioxidative and immune status. Food Funct..

[23] Wen W., Chen X., Huang Z., Chen D., Yu B., He J., Luo Y., Yan H., Chen H., Zheng P. (2022). Dietary lycopene supplementation improves meat quality, antioxidant capacity and skeletal muscle fiber type transformation in finishing pigs. Anim. Nutr..

[24] Shevchenko L., Iakubchak O., Davydovych V., Honchar V., Ciorga M., Hartung J., Kołacz R. (2021). Influence of lycopene and astaxanthin in feed on metabolic parameters of laying hens, yolk color of eggs and their content of carotenoids and vitamin A when stored under refrigerated conditions. Pol. J. Vet. Sci..

[25] Meng Q., Zhang Y., Li J., Shi B., Ma Q., Shan A. (2022). Lycopene affects intestinal barrier function and the gut microbiota in weaned piglets via antioxidant signaling regulation. J. Nutr..

[26] Papaioannou E.H., Liakopoulou-Kyriakides M., Karabelas A.J. (2016). Natural origin lycopene and its “green” downstream processing. Crit. Rev. Food Sci. Nutr..

[27] Grabowska M., Wawrzyniak D., Rolle K., Chomczyński P., Oziewicz S., Jurga S., Barciszewski J. (2019). Let food be your medicine: Nutraceutical properties of lycopene. Food Funct..

[28] Carvalho G.C., de Camargo B.A.F., de Araújo J.T.C., Chorilli M. (2021). Lycopene: From tomato to its nutraceutical use and its association with nanotechnology. Trends. Food Sci. Technol..

[29] Ikuyinminu E., Goñi O., O’Connell S. (2022). Enhancing irrigation salinity stress tolerance and increasing yield in tomato using a precision engineered protein hydrolysate and Ascophyllum nodosum-derived biostimulant. Agronomy.

[30] Ashraf W., Latif A., Lianfu Z., Jian Z., Chenqiang W., Rehman A., Hussain A., Siddiquy M., Karim A. (2022). Technological advancement in the processing of lycopene: A review. Food Rev. Int..

[31] Wang Y.-H., Zhang R.-R., Yin Y., Tan G.-F., Wang G.-L., Liu H., Zhuang J., Zhang J., Zhuang F.-Y., Xiong A.-S. (2022). Advances in engineering the production of the natural red pigment lycopene: A systematic review from a biotechnology perspective. J. Adv. Res..

[32] Li L., Liu Z., Jiang H., Mao X. (2020). Biotechnological production of lycopene by microorganisms. Appl. Microbiol. Biotechnol..

[33] Srivastava S., Srivastava A.K. (2015). Lycopene; chemistry, biosynthesis, metabolism and degradation under various abiotic parameters. J. Food Sci. Technol..

[34] Hedayati N., Naeini M.B., Nezami A., Hosseinzadeh H., Wallace Hayes A., Hosseini S., Imenshahidi M., Karimi G. (2019). Protective effect of lycopene against chemical and natural toxins: A review. BioFactors.

[35] Arballo J., Amengual J., Erdman J.W. (2021). Lycopene: A critical review of digestion, absorption, metabolism, and excretion. Antioxidants.

[36] Bin-Jumah M.N., Nadeem M.S., Gilani S.J., Mubeen B., Ullah I., Alzarea S.I., Ghoneim M.M., Alshehri S., Al-Abbasi F.A., Kazmi I. (2022). A natural arsenal in the war against oxidative stress and cardiovascular diseases. Antioxidants.

[37] Borel P. (2003). Factors affecting intestinal absorption of highly lipophilic food microconstituents (fat-soluble vitamins, carotenoids and phytosterols). Clin. Chem. Lab. Med..

[38] Kong K.W., Khoo H.E., Prasad K.N., Ismail A., Tan C.P., Rajab N.F. (2010). Revealing the power of the natural red pigment lycopene. Molecules.

[39] Zhao Y., Ma D.-X., Wang H.-G., Li M.-Z., Talukder M., Wang H.-R., Li J.-L. (2020). Lycopene prevents DEHP-induced liver lipid metabolism disorder by inhibiting the HIF-1α-induced PPARα/PPARγ/FXR/LXR system. J. Agric. Food Chem..

[40] Liu X., Lin X., Zhang S., Guo C., Li J., Mi Y., Zhang C. (2018). Lycopene ameliorates oxidative stress in the aging chicken ovary via activation of Nrf2/HO-1 pathway. Aging.

[41] Rajput S.A., Liang S.-J., Wang X.-Q., Yan H.-C. (2021). Lycopene protects intestinal epithelium from deoxynivalenol-induced oxidative damage via regulating Keap1/Nrf2 Signaling. Antioxidants.

[42] Fachinello M.R., Gasparino E., Partyka A.V.S., de Souza Khatlab A., Castilha L.D., Huepa L.M.D., Ferreira L.F.M., Pozza P.C. (2020). Dietary lycopene alters the expression of antioxidant enzymes and modulates the blood lipid profile of pigs. Anim. Prod. Sci..

[43] Fachinello M.R., Gasparino E., Monteiro A.N.T.R., Sangali C.P., Partyka A.V.S., Pozza P.C. (2020). Effects of dietary lycopene on the protection against oxidation of muscle and hepatic tissue in finishing pigs. Asian-Australas. J. Anim. Sci..

[44] Sun B., Ma J., Zhang J., Su L., Xie Q., Bi Y. (2014). Lycopene regulates production performance, antioxidant capacity, and biochemical parameters in breeding hens. Czech. J. Anim. Sci..

[45] An B.-K., Choo W.-D., Kang C.-W., Lee J., Lee K.-W. (2019). Effects of dietary lycopene or tomato paste on laying performance and serum lipids in laying hens and on malondialdehyde content in egg yolk upon storage. J. Poult. Sci..

[46] Al-Jrrah I.A., Abbas R.J. (2020). Effect of natural and synthetic sources of lycopene on productive performance, carcass quality and viscera relative weights of Japanese quail Coturnx japonica Temminck & Schlegel, 1849. Basrah. J. Agric. Sci..

[47] Amer S.A., Kishawy A.T., Osman A., Mahrose K.M., Hassanine E.-S.I., Rehman Z.U. (2020). Influence of dietary graded levels of lycopene on the growth performance, muscle cholesterol level and oxidative status of Japanese quail fed high-fat diet. An. Acad. Bras. Cienc..

[48] Wang S., Wu H., Zhu Y., Shi T., Cui H., Yang J., Lu M., Cheng H., Xu L. (2022). Effect of lycopene on the growth performance, antioxidant enzyme activity and expression of gene in Keap1-Nrf2 signaling pathway of Arbor Acres broilers. Front. Vet. Sci..

[49] Wan X., Yang Z., Ji H., Li N., Yang Z., Xu L., Yang H., Wang Z. (2021). Effects of lycopene on abdominal fat deposition, serum lipids levels and hepatic lipid metabolism-related enzymes in broiler chickens. Anim. Biosci..

[50] Mezbani A., Kavan B.P., Kiani A., Masouri B. (2019). Effect of dietary lycopene supplementation on growth performance, blood parameters and antioxidant enzymes status in broiler chickens. Livest. Res. Rural. Dev..

[51] Lee K.-W., Choo W.-D., Kang C.-W., An B.-K. (2016). Effect of lycopene on the copper-induced oxidation of low-density lipoprotein in broiler chickens. Springerplus.

[52] Wan X., Li N., Chen Y., Chen X., Yang Z., Xu L., Yang H., Wang Z. (2021). Protective effects of lycopene on mitochondrial oxidative injury and dysfunction in the liver of aflatoxin B1-exposed broilers. Poult. Sci..

[53] Sarker M.T., Wang Z.Y., Yang H., Wan X., Emmanuel A. (2021). Evaluation of the protective effect of lycopene on growth performance, intestinal morphology, and digestive enzyme activities of aflatoxinB1 challenged broilers. Anim. Sci. J..

[54] Sahin K., Orhan C., Tuzcu M., Sahin N., Hayirli A., Bilgili S., Kucuk O. (2016). Lycopene activates antioxidant enzymes and nuclear transcription factor systems in heat-stressed broilers. Poult. Sci..

[55] Pozzo L., Tarantola M., Biasibetti E., Teresa Capucchio M., Pagella M., Mellia E., Bergagna S., Gennero M.S., Strazzullo G., Schiavone A. (2013). Adverse effects in broiler chickens fed a high lycopene concentration supplemented diet. Can. J. Anim. Sci..

[56] Arain M.A., Mei Z., Hassan F., Saeed M., Alagawany M., Shar A., Rajput I. (2018). Lycopene: A natural antioxidant for prevention of heat-induced oxidative stress in poultry. Worlds Poult. Sci. J..

[57] Sahin K. (2015). Modulation of NF-κB and Nrf2 pathways by lycopene supplementation in heat-stressed poultry. Worlds Poult. Sci. J..

[58] Chen J., Tian M., Guan W., Wen T., Yang F., Chen F., Zhang S., Song J., Ren C., Zhang Y. (2019). Increasing selenium supplementation to a moderately-reduced energy and protein diet improves antioxidant status and meat quality without affecting growth performance in finishing pigs. J. Trace Elem. Med. Biol..

[59] Cartoni Mancinelli A., Mattioli S., Twining C., Dal Bosco A., Donoghue A.M., Arsi K., Angelucci E., Chiattelli D., Castellini C. (2022). Poultry meat and eggs as an alternative source of n-3 long-chain polyunsaturated fatty acids for human nutrition. Nutrients.

[60] Stampa E., Schipmann-Schwarze C., Hamm U. (2020). Consumer perceptions, preferences, and behavior regarding pasture-raised livestock products: A review. Food Qual. Prefer..

[61] An B.-K., Kim D.-H., Joo W.-D., Kang C.-W., Lee K.-W. (2019). Effects of lycopene and tomato paste on oxidative stability and fatty acid composition of fresh belly meat in finishing pigs. Ital. J. Anim. Sci..

[62] Rossi R., Pastorelli G., Cannata S., Tavaniello S., Maiorano G., Corino C. (2013). Effect of long term dietary supplementation with plant extract on carcass characteristics meat quality and oxidative stability in pork. Meat. Sci..

[63] Correia C.S., Alfaia C.M., Madeira M.S., Lopes P.A., Matos T.J.S., Cunha L.F., Prates J.A.M., Freire J.P.B. (2017). Dietary inclusion of tomato pomace improves meat oxidative stability of young pigs. J. Anim. Physiol. Anim. Nutr..

[64] Orhan C., Kucuk O., Sahin N., Tuzcu M., Sahin K. (2021). Lycopene supplementation does not change productive performance but lowers egg yolk cholesterol and gene expression of some cholesterol-related proteins in laying hens. Br. Poult. Sci..

[65] Hsu W.-T., Chiang C.-J., Chao Y.-P., Chang C.-H., Lin L.-J., Yu B., Lee T.-T. (2015). Effects of recombinant lycopene dietary supplement on the egg quality and blood characteristics of laying quails. J. Biosci. Bioeng..

[66] Sahin N., Akdemir F., Orhan C., Kucuk O., Hayirli A., Sahin K. (2008). Lycopene-enriched quail egg as functional food for humans. Food Res. Int..

[67] Kang H.-G., Lee S., Jeong P.-S., Kim M.J., Park S.-H., Joo Y.E., Park S.H., Song B.-S., Kim S.-U., Kim M.K. (2021). Lycopene improves in vitro development of porcine embryos by reducing oxidative stress and apoptosis. Antioxidants.

[68] Cao L., Zhao J., Ma L., Chen J., Xu J., Rahman S.U., Feng S., Li Y., Wu J., Wang X. (2021). Lycopene attenuates zearalenone-induced oxidative damage of piglet sertoli cells through the nuclear factor erythroid-2 related factor 2 signaling pathway. Ecotoxicol. Environ. Saf..

[69] Rawal S., Kim J.E., Coulombe R. (2010). Aflatoxin B1 in poultry: Toxicology, metabolism and prevention. Res. Vet. Sci..

[70] Wang X., Wang T., Nepovimova E., Long M., Wu W., Kuca K. (2022). Progress on the detoxification of aflatoxin B1 using natural anti-oxidants. Food Chem. Toxicol..

[71] Wan X., Ji H., Ma H., Yang Z., Li N., Chen X., Chen Y., Yang H., Wang Z. (2022). Lycopene alleviates aflatoxin B1 induced liver damage through inhibiting cytochrome 450 isozymes and improving detoxification and antioxidant systems in broiler chickens. Ital. J. Anim. Sci..

[72] El-Sheshtawy S.M., El-Zoghby A.F., Shawky N.A., Samak D.H. (2021). Aflatoxicosis in Pekin duckling and the effects of treatments with lycopene and silymarin. Vet. World.

[73] Sun B., Ma J., Zhang J., Su L., Xie Q., Gao Y., Zhu J., Shu D., Bi Y. (2014). Lycopene reduces the negative effects induced by lipopolysaccharide in breeding hens. Br. Poult. Sci..

[74] Davis E. (2022). Immunometabolism and inflammation: A perspective on animal productivity. Anim. Front..

[75] Lauridsen C. (2019). From oxidative stress to inflammation: Redox balance and immune system. Poult. Sci..

[76] Liu A., Chen X., Huang Z., Chen D., Yu B., Chen H., He J., Yan H., Zheng P., Yu J. (2022). Effects of dietary lycopene supplementation on intestinal morphology, antioxidant capability and inflammatory response in finishing pigs. Anim. Biotechnol..

[77] Fachinello M.R., Fernandes N.L.M., de Souto E.R., dos Santos T.C., da Costa A.E.R., Pozza P.C. (2018). Lycopene affects the immune responses of finishing pigs. Ital. J. Anim. Sci..

[78] Shevchenko L., Nedosekov V., Davydovych V., Rozhdestveskaya T., Drozdova E. (2021). Impact of lycopene and astaxanthin on hematological and immunological parameters of laying hens. IOP Conf. Ser. Earth Environ. Sci..

[79] Alwash I.J., Al–Khafaji F.R. (2018). Effect of adding different levels of lycopene to the diet on some physiological traits for Japanese quail bird exposed to thermal stress. Euphrates. J. Agric. Food Sci..

[80] Sarker M.T., Wan X., Yang H., Wang Z. (2021). Dietary lycopene supplementation could alleviate aflatoxin b1 induced intestinal damage through improving immune function and anti-oxidant capacity in broilers. Animals.

[81] Tian H., Liu G., Guo Y., Li Y., Deng M., Liu D., Sun B. (2020). Lycopene supplementation regulates the gene expression profile and fat metabolism of breeding hens. J. Anim. Physiol. Anim. Nutr..

[82] Guo X., Chen J., Yang J., He Q., Luo B., Lu Y., Zou T., Wang Z., You J. (2021). Seaweed polysaccharide mitigates intestinal barrier dysfunction induced by enterotoxigenic Escherichia coli through NF-κB pathway suppression in porcine intestinal epithelial cells. J. Anim. Physiol. Anim. Nutr..

[83] Gieryńska M., Szulc-Dąbrowska L., Struzik J., Mielcarska M.B., Gregorczyk-Zboroch K.P. (2022). Integrity of the intestinal barrier: The involvement of epithelial cells and microbiota—A mutual relationship. Animals.

[84] Chen J., Xia Y., Hu Y., Zhao X., You J., Zou T. (2023). A blend of formic acid, benzoic acid, and tributyrin alleviates ETEC K88-induced intestinal barrier dysfunction by regulating intestinal inflammation and gut microbiota in a murine model. Int. Immunopharmacol..

[85] Huting A.M.S., Middelkoop A., Guan X., Molist F. (2021). Using nutritional strategies to shape the gastro-intestinal tracts of suckling and weaned piglets. Animals.

[86] Tejeda O.J., Kim W.K. (2021). Role of dietary fiber in poultry nutrition. Animals.

